# Juvenile Nasopharyngeal Angiofibroma in Postmenopausal Females: A Potential Link With Hyperandrogenism

**DOI:** 10.7759/cureus.43256

**Published:** 2023-08-10

**Authors:** Song Ling Tang, Louis Luke, Salim Al-Shaikh

**Affiliations:** 1 Otolaryngology, James Paget University Hospitals NHS Foundation Trust, Great Yarmouth, GBR

**Keywords:** hyperandrogenism, nasal obstruction, otolaryngology, angiofibroma, juvenile nasopharyngeal angiofibroma

## Abstract

A 54-year-old female presented to the otolaryngology (ENT) outpatient department with an eight-month history of unilateral nasal obstruction and headache. There was no change in the sense of smell, rhinorrhoea, facial pain, or associated epistaxis. On examination, there was a large, erythematous mass in the superior aspect of the right nasal cavity, filling the space between the nasal septum, middle, and superior meatus. The rest of the ENT examination was normal. Vital signs were all within the normal range. There was no significant past medical history, and she had tried steroid nasal spray without any benefit. She had a complete resolution of symptoms from surgical intervention, and the mass was confirmed to be an angiofibroma through histopathology. This case report discusses the importance of considering nasopharyngeal angiofibroma as a differential diagnosis for patients presenting with unilateral nasal masses, including female patients, regardless of age.

## Introduction

Juvenile nasopharyngeal angiofibroma (JNA) is a rare, benign vascular tumor that usually occurs in adolescent males, often demonstrating aggressive features with local invasion [1,2]. There are also rare reports of older males and females [3]. It usually arises from the external carotid artery branches, with patients presenting with longstanding unilateral nasal obstruction, unprovoked epistaxis, and headaches [4]. Due to its anatomical location and vascularity, JNA can be difficult to treat surgically. We present a case of a 54-year-old female with right nasopharyngeal angiofibroma, a rare occurrence in both her age and gender group [5]. We also review similar cases reported in the literature to ascertain whether there may be an association with high androgen levels in post-menopausal women.

## Case presentation

A 54-year-old female presented to the ear, nose, and throat (ENT) outpatient department with unilateral nasal obstruction and headaches for the past eight months. She denied problems with her sense of smell, nasal discharge, facial pain, or epistaxis. Initially, she was managed in the primary care setting with steroid nasal spray. However, as she did not show any improvement, she was referred to ENT. She has no significant past medical history. On examination with rigid nasal endoscopy, a substantial erythematous mass was identified within the superior aspect of the right nasal cavity. It occupied the space between the middle and superior meatus and extended towards the nasal septum.

**Figure 1:**
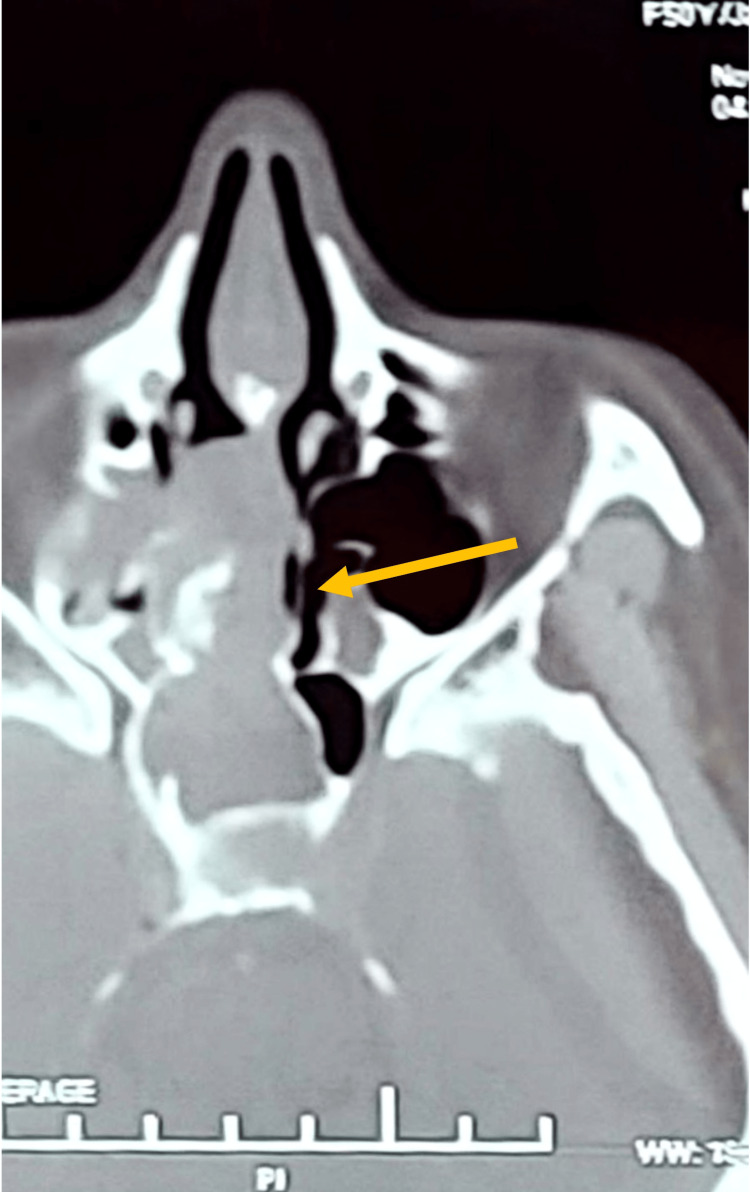
An axial view shows the mass completely occluding the right nasal cavity, resulting in a deviated nasal septum.

**Figure 2:**
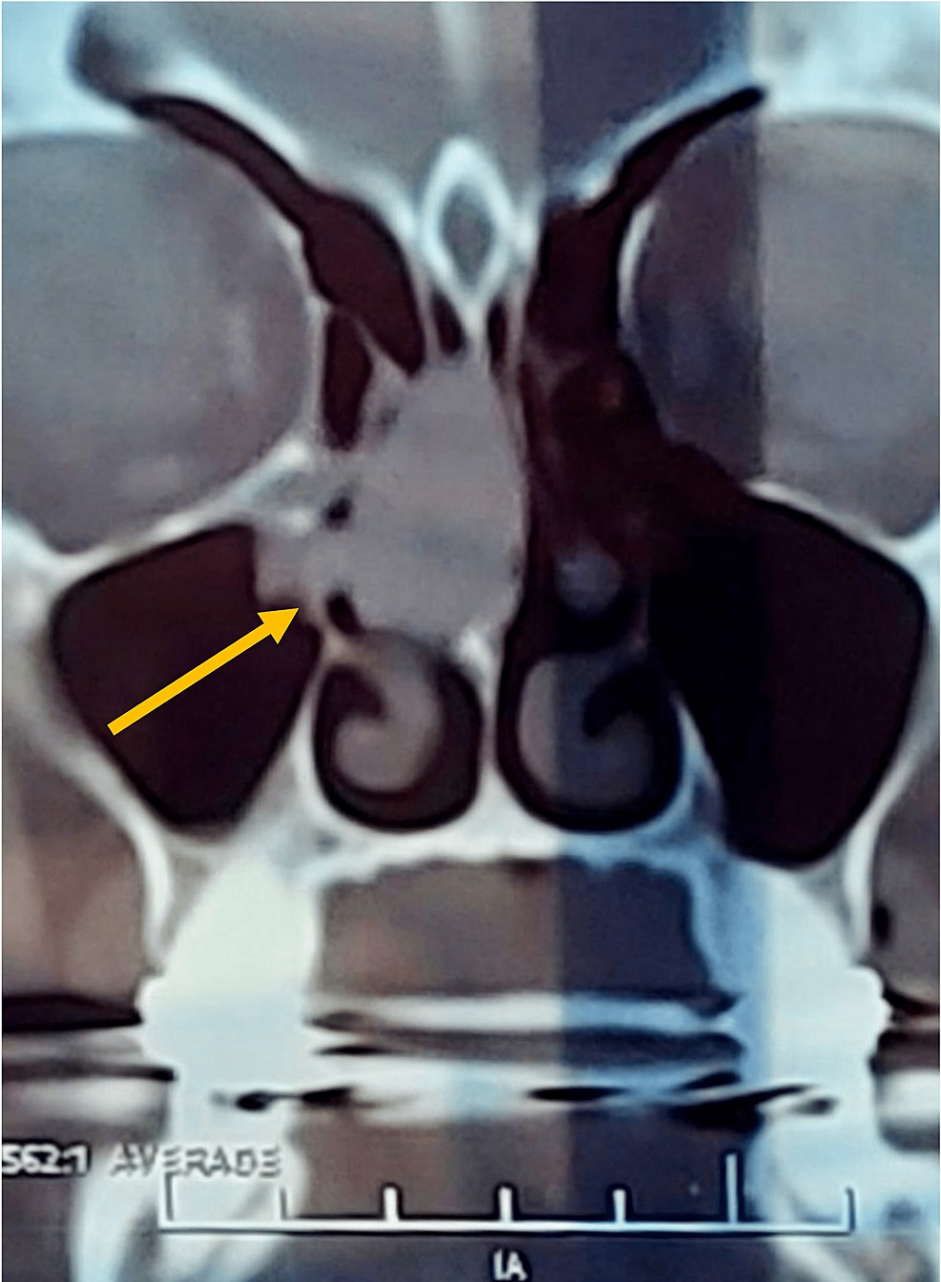
Coronal view showing the mass completely occluding the right nasal cavity, involving the right maxillary sinus ostium.

**Figure 3:**
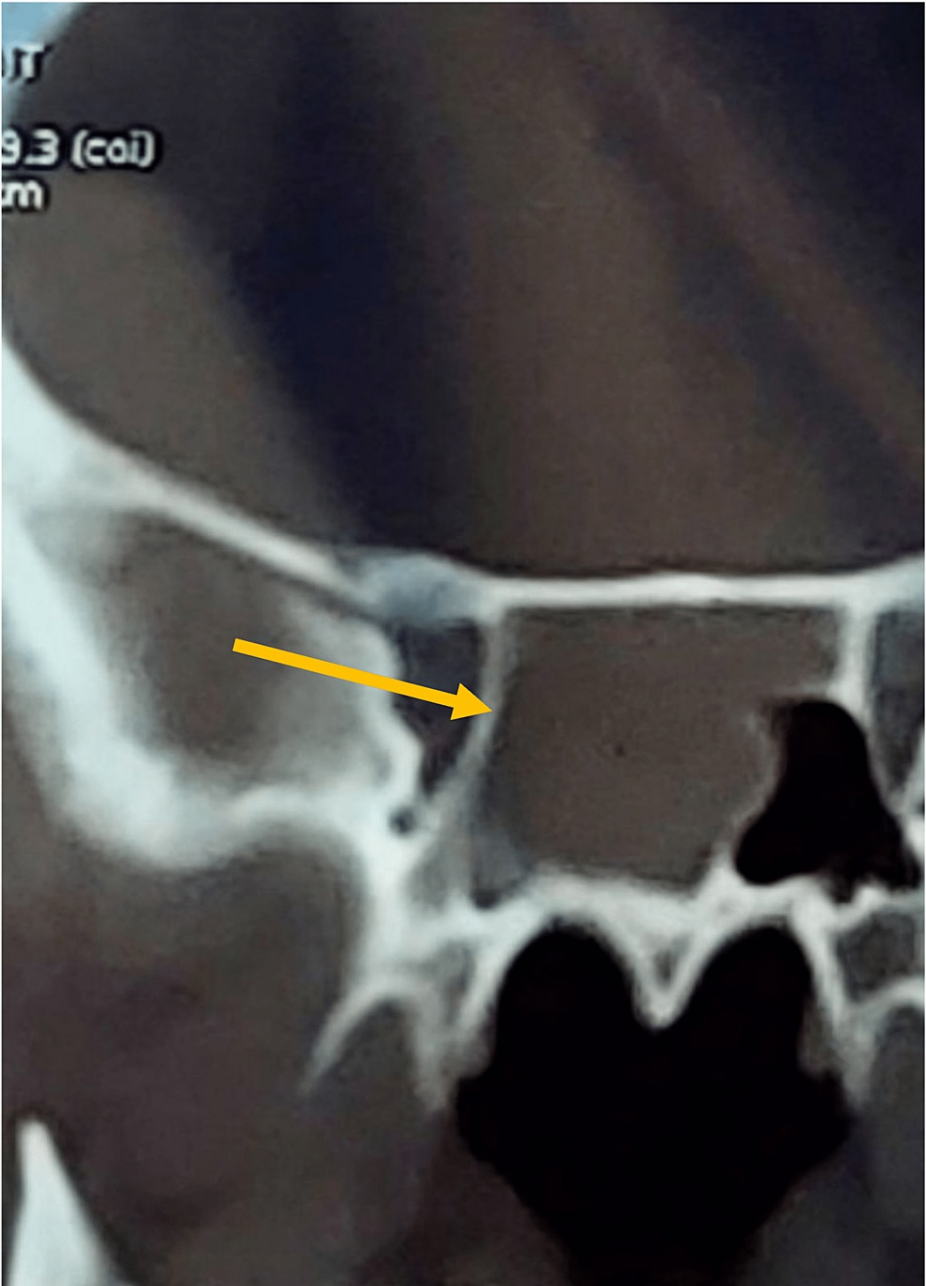
Coronal view showing the mass extending to the right sphenoid sinus, with a mass effect on the sphenoid sinus septation.

A computed tomography (CT) scan revealed a well-defined, homogeneous, enhancing mass that filled the right nasal cavity (Figure 1). The mass extended superiorly to the level of the cribriform plate and posteriorly into the nasopharynx, with complete opacification of the right sphenoid (Figures 2, 3). The remaining sinuses were clear. A biopsy was not performed preoperatively, as clinically, the lesion appeared to be an inverted papilloma, which required complete excision.

**Figure 4:**
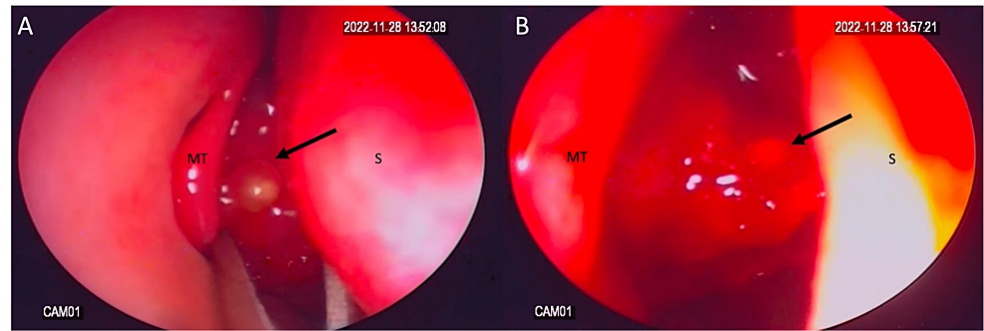
Endoscopic images of the right nasal cavity were acquired with a 0º endoscope. (A) Preoperative view of the angiofibroma (arrow) occluding the nasopharynx, with an anteriorly attached polypoid structure. (B) Intraoperative view of the angiofibroma (arrow) following partial resection. MT: middle turbinate; S: septum of the nose

The procedure was successful using an endoscopic approach, although there was some intraoperative bleeding (Figures 4A, 4B). To stop the bleeding, gelfoam was used at the end of the procedure, and ribbon gauze was inserted. The patient had a smooth recovery with no postoperative complications. The packing was removed two days later, and the patient used nasal saline douching for four weeks following the surgery.

The patient had follow-up appointments two weeks and four weeks after the operation, where the nasal fossa was examined with a rigid nasal endoscopy in the outpatient department. There were no remnants or recurrences of the mass found during the examination. The patient had another follow-up appointment scheduled four months later. During the appointment, the patient remained asymptomatic, and the examination was normal.

**Figure 5:**
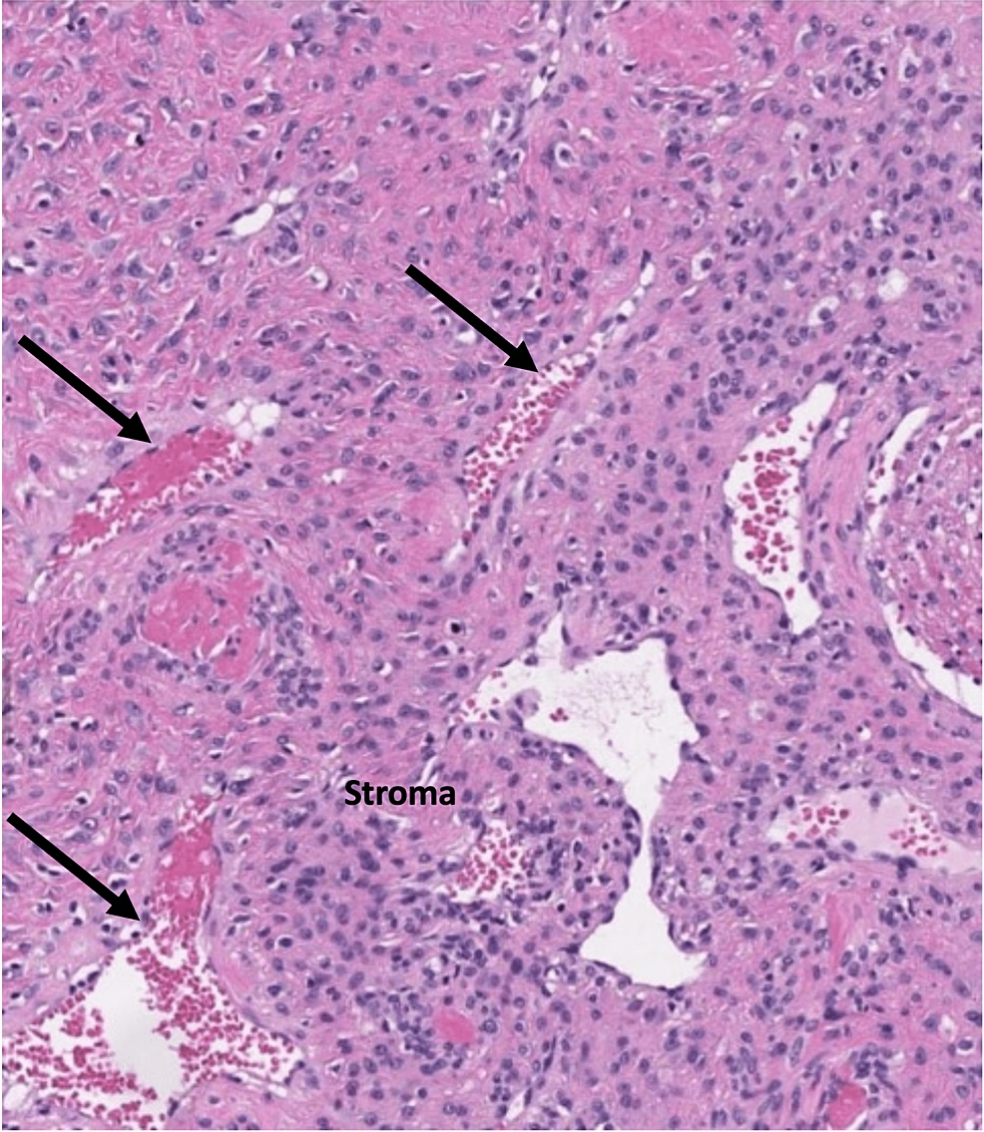
Micrograph of resected nasopharyngeal angiofibroma in hematoxylin-eosin (HE) stain, showing vascular space of various sizes (arrows) separated by fibrocellular stroma (labelled).

The diagnosis of nasopharyngeal angiofibroma was later confirmed by histopathology examination, which revealed vascular space of various sizes, ranging from dilated branching vessels of various thicknesses to slit-like capillaries. These were separated by fibrocellular stroma with fibroblasts (Figure 5).

## Discussion

JNA is a rare benign tumor affecting predominantly adolescent males, with the average age of presentation being 14 to 25 years old [5]. Some genetic studies hypothesized that its androgen-dependent nature is related to its origin-a vascular nidus within the sphenopalatine foramen-stimulated by endogenous androgenic hormones during early puberty [6,7]. During this time, coincident with the increase in testosterone levels, the tumor grows and becomes symptomatic.

JNA classically presents as a painless, progressive unilateral nasal obstruction, occasionally accompanied by epistaxis resulting from its highly vascular nature. Given our patient’s history, gender, and age group, we considered several differential diagnoses, including malignancy, hemangiopericytoma, and inverted papilloma.

Preoperative radiological imaging, such as CT, magnetic resonance imaging (MRI), and angiography, is valuable in assessing JNA [8]. The combination of clinical and radiological findings is usually sufficient for diagnosis; however, angiography would aid preoperative selective embolization to minimize blood loss. In our case, CT imaging was employed for tumor staging and detection of involvement of nearby bones and intracranial structures. Nonetheless, embolization was not performed due to a low initial suspicion of angiofibroma.

JNA is locally invasive and aggressive, likely attributed to its non-encapsulated and highly vascular nature. These factors contributed to a relatively high recurrence rate of 24.5% with an average follow-up period of 49 months, as reported in a recent meta-analysis by Reyes et al. [9]. In our case, there was no tumor recurrence after over a year of follow-up. Nonetheless, studies [9,10] have shown the use of an endoscopic approach to treat JNA (without intracranial involvement) has a significantly lower recurrence rate compared to the open surgical approach.

To date, there have been only a limited number of reported cases of JNA in females. Marcos et al. and Massimo et al. described two JNA cases in post-menopausal females (60 and 68 years old, respectively) [11,12]. Overall reported JNA cases in females are predominantly found in adolescent females [13,14]. During puberty, both genders experience increased androgen production. While androgen levels in adolescent females are lower than in their male counterparts, they are higher when compared to prepubertal girls. Adolescent females produce androgens in the adrenal glands and ovaries, which promote growth spurts and muscle mass development [15].

Post-menopause, the significant decline in estrogen production leads to a relative increase in the androgen-to-estrogen ratio. Linking to the occurrence of postmenopausal JNA-as observed in our case study and supported by existing reports [11,12]-we suggest that JNA in females may have naturally regressed during the post-pubertal years of heightened estrogen production but re-emerged after menopause due to decreased estrogen levels. Interestingly, Peloquin et al. have also reported a rare JNA case in a pregnant woman [16], which could potentially be correlated with the increase in androgen levels throughout gestation [17].

Given its rare incidence and association with elevated androgen levels, we recommend screening for causes of hyperandrogenism in female patients, e.g., medications and rare renal and ovarian androgen-secreting tumors. Detailed history-taking could help identify chronic conditions like polycystic ovarian syndrome (PCOS) and non-classic congenital adrenal hyperplasia (NCCAH). Further research is required to elucidate the relationship between JNA and androgen levels in females, understand its pathogenesis, and set directions for potential non-invasive interventions.

## Conclusions

JNA lacks a definite pathogenesis; however, studies and case reports suggest its association with hyperandrogenism. The uncommon occurrence in females highlights the need to consider JNA as a differential diagnosis in nasopharyngeal mass cases. Further research is required to understand the link between JNA and androgens across different gender and age groups.
